# A Case of Reed Syndrome with a Novel Mutation in the Fumarate Hydratase Gene

**DOI:** 10.1155/2013/926896

**Published:** 2013-07-09

**Authors:** Christin B. Laufer, Layne B. Green, Darren E. Whittemore

**Affiliations:** ^1^Department of Internal Medicine, Keesler Medical Center, Biloxi, MS 39534, USA; ^2^Department of Dermatology, San Antonio Uniformed Services Health Education Consortium (SAUSHEC), San Antonio, TX 78236, USA; ^3^Department of Dermatopathology, San Antonio Uniformed Services Health Education Consortium (SAUSHEC), San Antonio, TX 78236, USA

## Abstract

Reed syndrome is a heritable cancer predisposition syndrome that can easily be missed due to its simple presentation of tender red papules. We present a young female with a history of uterine fibroids who presented to the dermatology clinic with several painful pink papules that had been previously evaluated by multiple physicians. Biopsy results were diagnostic for cutaneous leiomyomas, raising clinical suspicion for Reed syndrome. She was found to have a novel heterozygote mutation in her fumarate hydratase gene, supporting the diagnosis. This case demonstrates the importance of rendering a proper workup for seemingly innocent skin complaints as they could be associated with an underlying malignancy. Despite the fact that up to 16% of patients can develop aggressive type 2 papillary renal cell carcinoma, there are currently no consensus guidelines on screening or patient management.

## 1. Case Presentation

**Figure 1:**
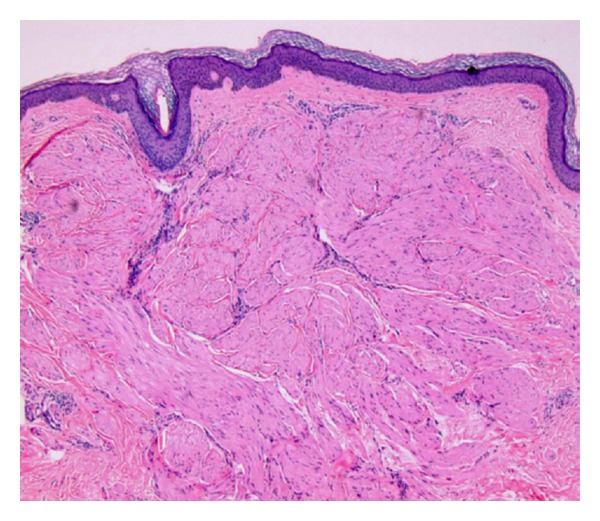
H&E 4x.

**Figure 2:**
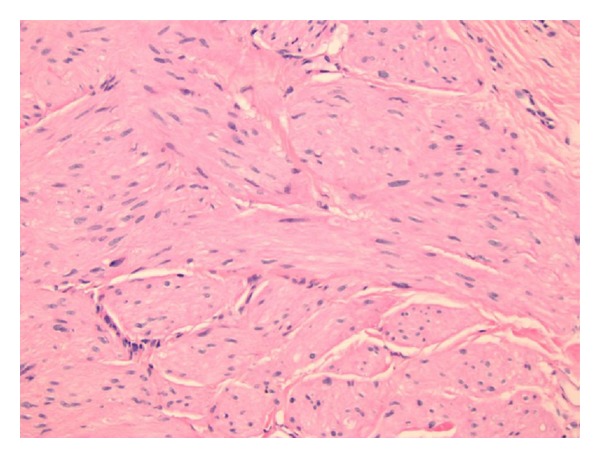
H&E 20x.

**Figure 3:**
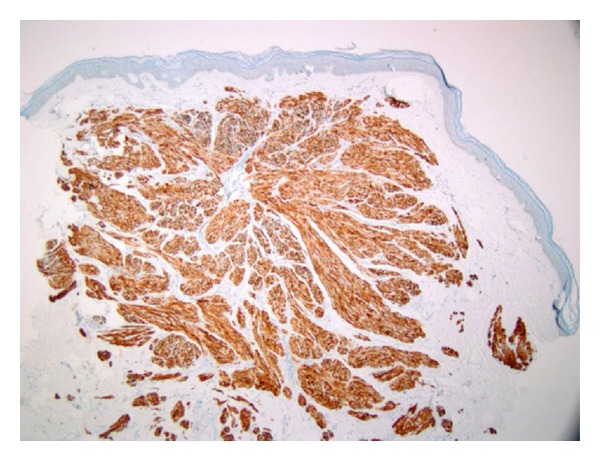
Desmin immunohistochemistry stain.

A 37-year-old Fitzpatrick type II German female presented with a chief complaint of “painful bumps” on her extremities which started 15 years ago. She had seen several primary care physicians and dermatologists in the past and had one “bump” excised in Germany but had never been offered a diagnosis and was unable to access her records. Past medical history was pertinent for multiple uterine leiomyomas for which she underwent a total abdominal hysterectomy (TAH) that revealed a uterine smooth muscle tumor of uncertain malignant potential (STUMP). Family history was remarkable for a sister who also had similar “bumps” on her extremities which had never been biopsied. There was no family history of renal malignancies. Medications, allergies, and other review of systems were noncontributory. Physical exam revealed tender pink to erythematous 3–6 mm firm dermal papules on her bilateral calves and right anterior tibialis in the center of a 1 cm scar where one of her previous papules had been excised. Three 4 mm punch biopsies of the new papules revealed uniform interlacing fascicles of dermal spindle-shaped cells with brightly eosinophilic cytoplasm, blunt-ended or “cigar-shaped” nuclei without significant pleomorphism, and lacking mitotic figures (Figures 1 and 2). By immunohistochemistry, the spindle cells were desmin positive and S-100 negative, consistent with a diagnosis of cutaneous piloleiomyomata (Figure 3). Her biopsy results in conjunction with her personal and family history raised our clinical suspicion for Reed syndrome. Sequencing of her fumarate hydratase gene was performed using a blood sample at Baylor Medical Center in Houston, Texas. Coding exons and the immediate flanking intronic nucleotides were sequenced using the Sanger dideoxy method. Sequencing revealed a novel loss of function, heterozygous missense mutation in exon 2 resulting in a G69V substitution, thus supporting the diagnosis. An MRI of her abdomen performed prior to her hysterectomy and a CT scan performed nine months after her hysterectomy revealed no evidence of metastatic disease from the STUMP or renal abnormalities.

## 2. Discussion

Reed syndrome is a rare hereditary cancer predisposition syndrome associated with multiple cutaneous and uterine leiomyomas and type 2 papillary renal cell carcinomas (pRCC) as well as collecting duct carcinomas [1]. Up to 16% of patients can develop a particularly aggressive type 2 pRCC [2]. It is due to a loss of function mutation in the fumarate hydratase (FH) gene on chromosome 1q42.3-q43 and can be inherited in an autosomal dominant fashion due to the mutation's dominant negative effect on the homotetrameric enzyme [1]. It can also develop de novo during embryologic development. Reed syndrome displays variable expressivity as a result of poorly understood epigenetic mechanisms that attenuate or accentuate gene expression. The pathogenesis of neoplasia formation can be described by Knudson's two-hit hypothesis in which there must be a loss of heterozygosity to be phenotypically affected.

Most patients are young to middle aged and present to their physician with complaints of new tender pink to red skin nodules or papules. Although most of the patients (approximately 76%) will have multiple or single cutaneous leiomyomas, a minority will present solely with uterine leiomyomas with or without an associated underlying renal malignancy [2]. Almost all female patients will have a history of uterine leiomyomas. They also may have a family history of similar findings in addition to renal malignancies. On physical exam, they can have isolated or innumerable firm papules that may contract in response to cold temperatures. Some patients exhibit genetic mosaicism where a mutation is present in only a subset of cells due to an error in DNA replication during embryologic development [3]. Such patients may exhibit a single cluster of cutaneous leiomyomata in close proximity to each other and often with a linear configuration following the lines of blaschko, which represents the mosaic cell line. Biopsy of the cutaneous lesions will reveal an ill-defined, somewhat circumscribed proliferation of spindle cells in bundles and fascicles with elongated nuclei and brightly eosinophilic cytoplasm. The definitive diagnosis of Reed syndrome can be made by detecting a mutation in the FH gene upon sequencing or by detecting decreased enzyme activity [2].

**Figure 4:**
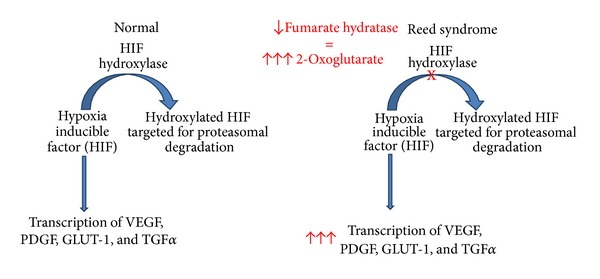
Pathogenesis.

Fumarate hydratase is the enzyme responsible for converting fumarate to malate in the tricyclic carboxylic acid cycle. Decreased expression of FH leads to a buildup of fumarate, succinate, and 2-oxoglutarate, as well as increased cellular dependence on glycolysis for ATP production. Fumarate hydratase acts as a tumor suppressor gene by regulating hypoxia inducible factor (HIF), which in increased quantities appears to be strongly associated with renal malignancies. HIF 1 and 2 alpha participate as transcription factors for multiple genes, including the protooncogenes vascular endothelial growth factor (VEGF), glucose transporter-1 (GLUT-1), platelet-derived growth factor (PDGF), and transforming growth factor alpha (TGF alpha) [4]. Normally, HIF prolyl hydroxylase hydroxylates HIF through an oxygen-mediated pathway, which allows it to be ubiquitinated by the Von Hippel-Lindau protein, targeting HIF for proteasomal degradation [5]. Under hypoxic conditions, HIF hydroxylase is unable to hydroxylate HIF. This results in elevated levels of HIF which activate expression of its downstream genes. Patients with Reed syndrome are thought to be in a pseudohypoxic state [6] in which a buildup of 2-oxoglutarate can competitively inhibit HIF hydroxylase for poorly understood reasons [5], thus ultimately decreasing proteasomal degradation of HIF and leading to upregulation of its downstream protooncogenes, promoting tumorigenesis [5] (Figure 4). 

The most important management aspect of patients diagnosed with Reed syndrome is detecting and treating associated renal cancer early; however, there are currently no consensus screening guidelines. Reed-syndrome-associated renal tumors classically have an aggressive clinical course and can arise with varying presentations at different ages, including childhood [2]. One proposed screening plan consists of the first renal ultrasound (US) and magnetic resonance imaging (MRI) starting at age 20, followed by annual MRI exams and semiannual US exams [1]. Computed tomography (CT) scans should be avoided to reduce any chance of provoking renal cancer by inducing a second hit [7, 8]. Additional management plans could be extrapolated from the National Comprehensive Cancer Network (NCCN) guidelines for more common hereditary cancer predisposition diseases such as Li-Fraumeni and Cowden syndrome. These guidelines recommend at a minimum an annual comprehensive, full physical examination with complete laboratory workup, patient education regarding signs and symptoms of cancer (hematuria, palpable abdominal mass, flank pain, and new onset hypertension), and target surveillance based on individual family histories [9].

When managing hereditary cancer predisposition syndromes, it is imperative that the physician takes ethical and social considerations into account. Our diagnosis for this patient inherently has implications on her family members, especially her sister who may have the same diagnosis. Since her detected mutation on sequencing was novel, referencing the Fumarate Hydratase gene in gene databases could not be used to predict cancer development [10]. However, this particular mutation was deemed to be pathologic at the time of sequencing based on the Sorting Intolerant from Tolerant (SIFT) and Polymorphism Phenotyping v2 (PolyPhen-2) algorithms. The former uses evolutionary conservation and sequence homology to make phenotype predictions while the latter predicts how an amino acid substitution will affect structure and therefore function of a protein. We therefore recommended to the patient that her parents undergo DNA sequence analysis to better understand the clinical significance. The patient was referred to a local geneticist and genetic counselor for consideration of the significance of this condition affecting her and her family members.

## 3. Conclusion

Reed syndrome is an uncommon cancer predisposition syndrome associated with an aggressive form of renal cell carcinoma that often presents as simple tender pink papules [2]. Although our patient did not have a renal malignancy present, her diagnosis had been missed by multiple physicians, demonstrating the importance of rendering an appropriate workup for seemingly innocent skin changes. Our management schema consists of baseline and annual renal imaging with US or MRI, patient education regarding the disease, and annual complete physical examinations and laboratory workups. The patient should additionally be referred to genetic counseling, where the pathogenicity of the underlying mutation can be predicted and where family members can receive proper care.
